# Status of Beverages Served to Young Children in Child Care After Implementation of California Policy, 2012–2016

**DOI:** 10.5888/pcd17.190296

**Published:** 2020-04-09

**Authors:** Danielle L. Lee, Klara Gurzo, Lilly A. Nhan, Elyse Homel Vitale, Sallie Yoshida, Ken Hecht, Lorrene D. Ritchie

**Affiliations:** 1 University of California, Division of Agriculture and Natural Resources, Nutrition Policy Institute, Berkeley, California; 2 Stockholm University, Department of Public Health Sciences, Stockholm, Sweden; 3 University of California, Los Angeles, Fielding School of Public Health, Community Health Sciences, Los Angeles, California; 4 California Food Policy Advocates, Oakland, California; 5 Child Care Food Program Roundtable, Los Angeles, California; 6 The Sarah Samuels Center for Public Health Research and Evaluation, Oakland, California

## Abstract

**Introduction:**

Since 2012, licensed California child care centers and homes, per state policy, are required to serve only unflavored low-fat or nonfat milk to children aged 2 years or older, no more than one serving of 100% juice daily, and no beverages with added sweeteners, and they are required to ensure that drinking water is readily accessible throughout the day. We evaluated adherence to the policy after 4 years in comparison to the adherence evaluation conducted shortly after the policy went into effect.

**Methods:**

Licensed California child care sites were randomly selected in 2012 and 2016 and surveyed about beverage practices and provisions to children aged 1–5 years. We used logistic regression to analyze between-year differences for all sites combined and within-year differences by site type and participation in the federal Child and Adult Care Food Program (CACFP) in self-reported policy adherence and beverage provisions.

**Results:**

Respondents in 2016 (n = 680), compared with those in 2012 (n = 435), were more adherent to California’s 2010 Healthy Beverages in Child Care Act overall (45.1% vs 27.2%, *P* < .001) and with individual provisions for milk (65.0% vs 41.4%, *P* < .001), 100% juice (91.2% vs 81.5%, *P* < .001), and sugar-sweetened beverages (97.4% vs 93.4%, *P* = .006). In 2016, centers compared with homes (48.5% vs 28.0%, *P* = .001) and CACFP sites compared with non-CACFP sites (51.6% vs 27.9%, *P* < .001) were more adherent to AB2084 overall.

**Discussion:**

Beverage policy adherence in California child care has improved since 2012 and is higher in CACFP sites and centers. Additional policy promotion and implementation support is encouraged for non-CACFP sites and homes. Other states should consider adopting such policies.

**Summary**

**What is already known on this topic?**

In 2012, California enacted a policy designating that licensed child care sites serve only healthy beverages. A study conducted later that year showed positive changes. However, only 60% of child care survey respondents knew about the policy and only one-quarter were fully adherent.

**What is added by this report?**

We assessed the degree of adherence to the policy in 2016, finding continued improvements since 2012 and few implementation barriers reported.

**What are the implications for public health practice?**

Comprehensive state policy on beverages in child care can be successfully implemented and should be considered by other states.

## Introduction

Nearly 1 in 7 children aged 2–5 years in the United States is obese (1), and sugary beverage consumption is a major contributing factor to excessive weight gain in young children (2,3). Child care sites are an ideal setting for improving the quality of beverages consumed (4); nearly two-thirds of US children younger than 5 years receive much of their daily nutrition in nonparental care settings (5).

Beginning in late 2016, licensed child care centers and homes participating in the federal Child and Adult Care Food Program (CACFP) were required to make readily available and actively offer drinking water throughout the day (6) and, beginning in late 2017, serve no more than 1 age-appropriate serving of 100% juice daily and unflavored low-fat or nonfat milk to children 2 years and older (7). Some states, including California, require licensed centers to follow CACFP standards, regardless of program participation (8). In addition, several states have adopted their own regulations on beverages served in child care, regardless of CACFP participation (9). For example, California’s 2010 Healthy Beverages in Child Care Act (AB2084), which went into effect in January 2012, requires the same provisions as CACFP for water, milk, and juice (only unflavored milk, 100% juice, and water served). In addition, AB2084 prohibits serving beverages with added natural or artificial sweeteners, such as soda and fruit drinks (10).

In 2012, several months after AB2084 went into effect, beverage provisions were evaluated in a statewide sample of licensed child care centers and homes in California (11,12). Only 60% of child care providers knew about the policy and about one-quarter of sites were fully adherent to the policy. We aimed to evaluate whether adherence has changed since 2012 by comparing to new data collected from licensed child care sites in California in 2016.

## Methods

This study was a cross-sectional assessment comparing independent samples of licensed child care centers (hereinafter referred to as “centers”) and family child care homes (hereinafter referred to as “homes”) surveyed in 2012 and 2016. Methods used in 2016 were similar to those used in 2012, as described previously (11,12). The study was deemed exempt by the Committee for the Protection of Human Subjects at the University of California, Davis.

### Sample selection and survey instrument

We used California Department of Social Services and Department of Education databases to identify all licensed centers and homes (approximately 43,000). Sites were stratified into 6 categories: 1) Head Start centers (required to participate in CACFP), 2) state preschools (participate in CACFP or follow federal school meal program guidelines that meet or exceed those of CACFP), 3) other centers participating and 4) not participating in CACFP, and 5) homes participating and 6) not participating in CACFP. A sample of 2,400 sites equivalently distributed by category were randomly selected to participate. 

The survey was based on a validated instrument (13) and was previously pilot-tested with minor modifications specific to study objectives. Questions were included about site characteristics as was a frequency checklist of beverage provisions the day before completing the survey for meals (breakfast, lunch, and dinner) and snacks served to young children (aged 2–5 in 2012, aged 1–5 in 2016). Respondents were instructed to report on beverages provided by the site and brought by parents, including those used for celebrations. Additional questions were asked about type of milk provided, how water was provided at meals/snacks, and facilitators and barriers to limiting fruit juice and providing only unflavored low-fat or fat-free milk.

### Sample recruitment and data collection

In fall 2016, all selected child care sites were mailed a postcard in English and Spanish with a link to the survey online (Qualtrics, version 08–2016, 2016); an email also was sent to sites with email addresses (n = 1,248). Paper surveys (in English for centers and both Spanish and English for homes) were mailed to nonrespondents 2 months later. When the response rate by child care category was less than 10%, nonresponders were contacted by telephone to encourage survey completion. A $5 gift card also was provided as an incentive.

### Data analysis

Data from paper surveys were double-entered to ensure accuracy, and online survey data (n = 155) were merged with the paper survey data (n = 581). Twenty surveys were excluded from analysis due to duplication (ie, completed on paper and online, in which case the first survey received was used) for an overall response rate of 30% (similar to the 31% achieved in 2012 [12]). Respondents to the 2016 survey were similar to the 2,400 randomly selected child care sites in terms of geography (mean difference in percentage by county of 0.3% [standard deviation (SD), 0.6%]) and whether they were at a center or home (mean, 6.0% [SD, 0.1%]). Surveys with more than 60% of incomplete questions on site characteristics were excluded (n = 36). Of the 680 surveys included in the 2016 analytical sample, 564 were from centers and 116 from homes. In 2012 and 2016, 12% and 11% of homes completed the survey in Spanish. From 2%–17% and 2%–9% of data were missing for survey items that assessed beverage practices and policy adherence, respectively, in 2012 and 2016. Imputation of missing data was not performed, and nonresponses were not included in the denominator when calculating percentage adherence to the policy.

Data were analyzed by using SAS version 9.4 (SAS Institute, Inc). Differences between survey respondent characteristics in 2012 and 2016 were assessed by using χ^2^ tests or Fisher exact tests (when cell sizes were small) for categorical variables and *t* tests for continuous variables. Binary variables on beverages (provided or not) and adherence to AB2084 (adherent or not) were created. Differences between beverages provided and adherence to AB2084 from 2012 to 2016 were assessed by using logistic regression models adjusted for CACFP status and whether the site was a center or home. The difference between CACFP participation was analyzed by using logistic regression adjusted for the site being center or home; difference between center and home was analyzed by using logistic regression adjusted for CACFP versus non-CACFP participant. Logistic regression was used to compare beverage practices and adherence to AB2084 in 2016 for the 6 types of child care categories and to conduct follow-up pairwise comparisons between the categories. Multiple comparisons were adjusted using the Bonferroni-Holm approach. *P* values < .05 were considered significant.

## Results

### Site characteristics

In 2012 and 2016, most survey respondents were the child care site director or owner, and most sites had been in operation for 5 or more years, provided full-day care (as opposed to only providing care for part of the day), and provided all foods and beverages served to young children. Between 2012 and 2016, there were small but significant differences in the proportion of sites in each child care category and providing full-day care (Table 1).

**Table 1:** Characteristics of California Child Care Sites Participating in the 2012 and 2016 Surveys on Beverages Served to Young Children Following Implementation of the Healthy Beverages in Child Care Act^a^

Characteristic	2012^b^ (n = 435)	2016 (n = 680)	*P* Value^c^
**Child care category^d^**			
Head Start	78 (17.9)	96 (14.1)	<.001
State preschool^e^	93 (21.4)	132 (19.4)	
CACFP center	48 (11.0)	183 (26.9)	
Non-CACFP center	82 (18.9)	153 (22.5)	
CACFP family home	93 (21.4)	68 (10.0)	
Non-CACFP family home	41 (9.4)	48 (7.1)	
**Child care site characteristic**			
Mean no. (SD) of children aged 2–5	81.9 (255.4)	74.1 (171.8)	.58
Mean no. (SD) of staff	15.7 (54.9)	14.8 (23.0)	.75
Site director/owner responded to survey	367 (85.3)	597 (89.2)	.06
Full-day care provided	310 (72.4)	543 (81.7)	<.001
**Years in operation^f^**			
<12 months	7 (1.6)	7 (1.0)	.11
1 to 5 years	60 (13.8)	68 (10.2)	
≥5 years	367 (84.6)	597 (88.8)	
**Source of foods and beverages**			
Site only	366 (84.5)	568 (84.6)	.55
Site and parents	46 (10.6)	78 (11.6)	
Parents only	7 (1.6)	12 (1.8)	
**Mean no. (SD) of meals and snacks served per day **			
Breakfast	316 (76.7)	488 (76.6)	.97
Lunch	336 (80.2)	537 (82.1)	.43
Dinner	84 (33.1)	78 (16.8)	<.001
Morning snack	211 (70.6)	305 (57.8)	<.001
Afternoon snack	322 (87.3)	534 (85.9)	.53
Evening snack	51 (23.4)	96 (21.4)	.57

Abbreviations: CACFP, Child and Adult Care Food Program; SD, standard deviation.

a Sample size may vary because of missing survey responses; approximately 0%–50% of data were missing depending on characteristic and survey year. The 2012 survey applied to children aged 2–5; the 2016 survey applied to children aged 1–5. Values are no. (%) unless otherwise indicated.

b Findings from the 2012 survey were published previously (11); values may differ slightly because of variations in coding methods used, handling of missing values, and rounding.

c Analyzed by χ^2^ tests for categorical variables and *t* tests for continuous variables.

d Head Start centers and state preschools are required to participate in CACFP or the federal school meals program. CACFP centers were other centers that also participated in CACFP.

e Refers to schools that participate in CACFP or follow federal school meal program guidelines that meet or exceed those of CACFP.

f Fisher exact test used instead of χ^2^ test because of small cell sizes.

### Adherence to beverage policy

Milk was provided the day before the survey by most sites (>90%) in both study years (Table 2). In 2016 compared with 2012, of the types of milk most often provided, significantly fewer sites proided 2% milk and significantly more provided 1% and rice or soy milk to children aged 2 years or older. Additionally, of the types of milk ever provided, significantly fewer sites ever provided whole, 2%, rice or soy, or flavored or sweetened milk in 2016 compared with 2012. Only 2.2% of sites ever provided flavored or sweetened milk in 2016, down from 7.7% in 2012. 

**Table 2:** Beverages Provided by California Child Care Sites Several Months (in 2012) and Several Years (in 2016) After Beverage Policy Based on the Healthy Beverages in Child Care Act Was Enacted^a^

Beverage	2012^b^ (n = 435)	2016 (n = 680)	*P* Value^c^
	No. (%)		
**Milk provided (any type) on day before survey**	389 (92.2)	605 (93.2)	.36
**Type most often provided**			
Whole	37 (8.9)	36 (5.8)	.15
2%	113 (27.2)	131 (21.1)	.006
1%	232 (55.8)	421 (67.8)	<.001
Nonfat	30 (7.2)	51 (8.2)	.62
Rice or soy	2 (0.5)	29 (4.7)	.003
Flavored or sweetened	2 (0.5)	4 (0.6)	.79
**Types of milk ever provided**			
Whole	117 (28.0)	62 (9.7)	<.001
2%	158 (37.8)	152 (23.8)	<.001
1%	269 (64.4)	436 (68.2)	.08
Nonfat	57 (13.6)	78 (12.2)	.37
Rice or soy	122 (29.2)	144 (22.5)	.002
Flavored or sweetened	32 (7.7)	14 (2.2)	<.001
**Water^d^**			
Available for self-serve indoors	333 (77.8)	546 (84.1)	.17
Available for self-serve outdoors	341 (79.9)	542 (83.0)	.58
Provided at the table with meals and snacks	57 (13.3)	81 (12.6)	.79
**100% Juice provided on day before survey^e^**	233 (58.3)	249 (38.4)	<.001
**Sugar-sweetened beverages provided on day before survey^f^**	26 (6.6)	17 (2.6)	.006

a Sample size may vary due to missing survey responses; 2%–9% of data were missing depending on the beverage and survey year. For 2012 survey, beverages were assessed for children aged 2–5; for 2016 survey, beverages were assessed for children aged 1–5.

b Findings from the 2012 survey were published previously (11); values may differ slightly due to variations in coding methods used, handling of missing values, and rounding.

c Analyzed by logistic regression models and adjusted for CACFP status and whether the site was a center or home.

d Includes bottled and tap water.

e Excludes fruit-flavored drinks including lemonade and aguas frescas.

f Includes soda, sports drinks, lemonade, fruit drinks, aguas frescas, and sweet teas. Does not include diet drinks sweetened with artificial sweeteners and no- or low-calorie natural sweeteners.

There were no significant differences between 2012 and 2016 in the proportion of sites making water available for self-serve indoors or outdoors. The proportion of sites providing water at the table with meals and snacks also did not change between 2012 and 2016. Both 100% juice and sugar-sweetened beverages (SSBs) were provided by significantly fewer sites the day before the survey in 2016 compared with 2012.

**Figure 1:**
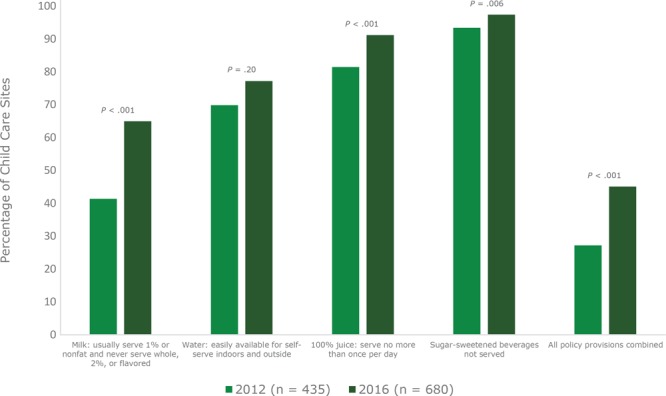
Adherence to policy by California child care sites several months (in 2012) and several years (in 2016) after beverage policies for young children were enacted. Data on policy adherence were collected from 2012 and 2016 surveys of California licensed child care providers.

In 2016, sites overall were near full adherence to the beverage provision that restricts serving SSBs (97.4%) and require serving no more than 1 serving of 100% juice (91.2%) (Figure 1).

**Table:** 

Policy Provision	2012 (n = 435)	2016 (n = 680)	*P* Value
Milk: usually serve 1% or nonfat and never serve whole, 2%, or flavored	41.4	65.0	<.001
Water: easily available for self-serve indoors and outside	69.9	77.2	.20
100% juice: serve no more than once per day	81.5	91.2	<.001
Sugar-sweetened beverages: not served	93.4	97.4	.006
All policy provisions combined	27.2	45.1	<.001

**Figure 2:**
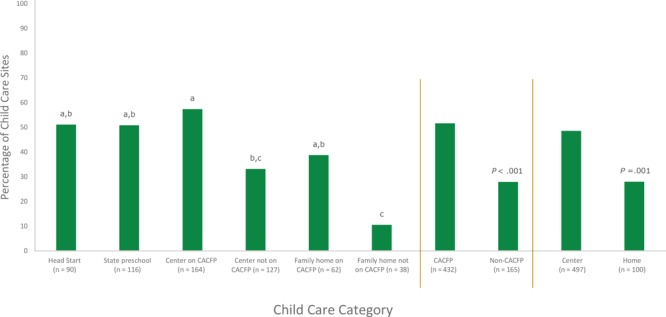
Adherence to all provisions of the California beverage policy, by type of child care in 2016 (n = 597). Data were collected from 2012 and 2016 surveys of California licensed child care providers. Because responses were missing for 1 or more beverages served to children aged 1–5 years, 597 sites in 2016 were assessed for full adherence to beverage policies. Results determined from pairwise comparisons. Values not sharing the same letter were significantly different from each other at *P* < .05 after applying the Bonferroni-Holm adjustment for multiple comparisons. Abbreviation: CACFP, Child and Adult Care Food Program.

Overall adherence to all 4 beverage provisions increased significantly to nearly half of sites (45.1%) in 2016 compared with approximately one-quarter of sites (27.2%) in 2012 (Figure 1), and significant improvements were also seen with individual provisions for milk (65.0% vs 41.4%, *P* < .001), 100% juice (91.2% vs 81.5%, *P* < .001), and SSBs (97.4% vs 93.4%, *P* = .006). In 2016, overall adherence was significantly higher for CACFP sites (51.6% of sites were adherent) compared with non-CACFP sites (27.9%), and for centers (48.5%) compared with homes (28.0%) (Figure 2).

**Table:** 

Child care type	Percentage of Child Care Sites
**Child care category**	
Head Start (n = 90)	51.1^a,b^
State preschool (n = 116)	50.8^a,b^
Center on CACFP (n = 164)	57.3^a^
Center not on CACFP (n = 127)	33.1^b,c^
Family home on CACFP (n = 62)	38.7^a,b^
Family home not on CACFP (n = 38)	10.5^c^
**CACFP Participation**	
CACFP (n = 432)	51.6
Non-CACFP (n = 165)	27.9 (*P* < .001)
**Child care centers and family child care homes**	
Center (n = 497)	48.5
Home (n = 100)	28.0 (*P* = .001)

Significant differences existed by child care category as well, with Head Start, state preschools, and other centers on CACFP having the highest proportion of sites adherent, and non-CACFP homes having the lowest proportion adherent.

### Factors influencing adherence to provisions on juice and milk

Most sites reported that limiting juice was not challenging (82.5%). However, the barriers to limiting juice that were most commonly reported included child preference (9.5%), parent preference or practice (9.3%), it not being a priority for the provider (2.8%), and other reasons (3.3%), such as commenting that juice is reimbursed by CACFP. Facilitators to limiting juice included policy or written guidelines (26.4%), information for families (22.3%), support from parents or families (21.6%), and training for child care providers (13.9%).

Most sites reported that serving only unflavored low-fat or fat-free milk to children aged 2–5 was not hard (88.4%). Reported barriers included child preference (4.9%), parent preference or practice (3.8%), it not being a priority for the provider (0.6%), or other reasons (2.6%), such as high cost or unavailability in places where food is purchased. Facilitators to providing only unflavored low-fat or fat-free milk to children aged 2–5 included policy or written guidelines (27.6%), support from parents or families (19.5%), information for families (19.4%), and training for child care providers (15.3%).

## Discussion

Improvements in adherence to providing healthy beverages in licensed child care sites were observed between 2012 and 2016 in California, 4 years after implementation of AB2084. Despite positive changes in beverage provisions since the law was enacted in 2012, additional room for improvement remains. More than half of sites were not adherent to all 4 beverage provisions, one-quarter did not make drinking water easily accessible throughout the day, and one-third were not adherent to the provision for milk. Provision of 2% milk (instead of 1% or nonfat) to children aged 2 years or older was the reason that most sites failed to be adherent to the milk policy. It is noteworthy that only 2% of California child care sites reported ever providing flavored or sweetened milk in 2016 to children aged 1–5 years. In comparison, more than 90% of US elementary schools serve flavored milk in grades K-12 (14).

New York City has a child care beverage policy similar to California, except that flavored milk and juice are not addressed. Compared with our 2016 California survey, in a 2010 study, adherence to the New York City policy was higher for milk (80%), and lower for SSBs (70%) and water (56%) (15). However, the New York City study only included centers, 90% of which participated in CACFP. Additionally, adherence in that study was assessed by observing beverage inventories of child care center facilities and classroom observations of beverages provisions during meals and snacks as opposed to self-report of beverages provided in our survey. These may contribute to differences between studies in adherence to beverage policy. In 2014 in Pennsylvania, where no state policy on beverages in child care exists, less than half (46%) of child care centers representing 88% of Pennsylvania counties answered no to the survey question “Are sugar-sweetened beverages provided?” (16). The lack of a policy in addition to the difference in survey question used to assess providing SSBs in the Pennsylvania study compared with our 2016 survey may have resulted in differences in reported SSBs provided. A more recent representative sample of child care centers and homes was assessed in 2017 in Georgia, where no state policy on child care beveverages exists, using the same questions as our California survey. In this study, most child care sites (96% in CACFP and 90% in non-CACFP) did not serve any SSBs on the day before being surveyed (17). It is noteworthy that in Georgia, fewer sites made water available for self-serve indoors (53%) and outdoors (41%), than in our California sample (84% and 83%, respectively). We are not aware of trend data on SSBs served in child care sites in other states that are comparable to our California data. However, growing awareness of the importance of healthy beverages for young children may have contributed to improved beverage servings practices among child care providers independent of policy. After increases during the end of the twentieth century (18), daily SSB intake by US children has declined in recent years. For example, in 2013 and 2014, 46.5% of children aged 2 to 5 years consumed an SSB on a given day, down from 68.9% a decade earlier (19).

Efforts to increase adherence to beverage policy should address provisions for milk and drinking water (adherence was lowest for serving only low-fat or non-fat milk to children 2 years or older) and ensuring water is easily available for self-serve both indoors and outdoors. Although few sites reported that the milk provision was challenging to implement, the barriers to implementation most often reported were child or parent preference. Policy or written guidelines were most often reported as facilitators of following the beverage policy on milk, which validates the need for state or federal policy. Support from parents or families and training for child care providers were also frequently reported. Although barriers and facilitators to implementation of the water policy were not included in this iteration of the child care survey, more than 75% of respondents in 2012 reported no barriers to making drinking water easily available throughout the day (11). Among those reporting barriers, sites participating in CACFP reported not getting any reimbursement from CACFP for offering water and a perception that CACFP requires that water not be served at meals and snacks (although no such CACFP regulation exists) (11). Given that the updated CACFP requirements include policy on water, milk, and 100% juice consistent with the California policy and that these federal requirements went into effect the year after our 2016 survey, follow-up assessments are warranted to examine whether adherence to the water and milk policies in California have since improved.

CACFP sites had higher adherence than non-CACFP sites with beverage policy, similar to other studies showing that CACFP participants provide better nutrition than those not participating in CACFP (20–23). CACFP sites receive more technical assistance and monitoring regarding required beverage policy than do child care sites that do not participate in the program. CACFP meal and snack reimbursements also may play a role in incentivizing healthier beverage provisions. In addition, it is possible that CACFP sites were more likely to be adherent to the California law in anticipation of the CACFP changes to requirements for water, milk, and 100% juice. Additionally, centers had higher adherence than homes, which may be due in part to the fact that California requires centers to meet CACFP nutrition standards regardless of program participation. Previous studies suggest that centers are often more adherent to nutrition policy than homes (21,24). Therefore, education and resources should target non-CACFP sites and homes to support improved beverage policy implementation for all young children in child care.

California licensed child care providers are informed of relevant state and federal requirements governing their operations in several ways. All licensed sites are required to stay up to date with policy, which are posted on the California Department of Social Services (CDSS) website (25). As of January 2016, all newly licensed child care sites are additionally required to participate in 1 hour of nutrition training, which includes information on AB2084 (26), per California’s 2013 Foundations for Healthy Nutrition in Child Care Act (27). However, less than 1% of survey respondents in the present study received their license after January 2016. Enforcement of policy is governed by the CDSS Community Care Licensing Division, which conducts facility evaluations for all licensed child care centers (28) and homes (29) once every 3 years. If a site is noncompliant, a citation may be issued based on a violation of the law; repeat violations warrant a civil penalty or fine. Providing additional guidance on AB2084 during licensing evaluations may be a cost-effective way to improve beverage nutrition in licensed child care.

As adherence to state and federal regulations regarding allowable beverages in California child care sites continues to improve, consumption of unhealthy beverages is likely to decrease, there being a strong and direct relationship between beverages served at child care sites and children’s beverage consumption (15). This may result both in long-term health care cost savings through preventing childhood obesity and possible cost savings for the child care site. Using simulation modeling, Wright et al found that policies in child care settings for restricting SSBs and only serving lower-fat milk, in addition to policies to reduce screen time and increase children’s physical activity, could result in a net health care cost savings per child of $6.15 per dollar spent administering the policy (30). That study also suggests that beverage policies can result in cost savings of $9.99 and $44.30 per year per child care site, on milk and SSBs, respectively (30). Further research is necessary to evaluate child beverage consumption at California child care sites and a nonsimulated cost–benefit analysis should be conducted.

This study has many strengths. Our study is the first that we know of to report data trends in providing beverages in child care in a state with a child care beverage policy. Additionally, compared with surveys used in other studies, our survey included multiple questions on types of beverages provided, clearly defined SSBs, and specified child age (16). Furthermore, it provided data from a large representative sample of California licensed child care providers of multiple child care site types, including family child care homes and CACFP and non-CACFP participants.

This study has several limitations. Even though consistent with other child care studies (11,16,31), the response rate was low for both survey years. Although sites were randomly selected, differences may exist between respondents and nonrespondents, and the sample may not be representative of California child care. In addition, results are based on self-report and sites may have reported desirable practices instead of actual practices. Analogous to how a 24-hour dietary recall is used for surveillance of the intakes of a population of individuals, for juice and SSB provisions, adherence in child care sites was based on beverages provided on a single day. However, assessing a single day may not fully capture long-term beverage practices. Given the observational nature of the study design, causality cannot be inferred; the improvements in beverage provisions from 2012 to 2016 may have resulted at least in part from secular trends in awareness of healthy beverages. Because all licensed sites in California are subject to the state policy, we were unable to include a control group. Finally, although licensed child care in California represents one-eighth of the total sites in the nation (32), our findings cannot be used to infer current practice in license-exempt or unlicensed sites and sites outside of the state.

To our knowledge, this is the first study to show continued improvement in adherence several years after the implementation of state policy mandating that only healthy beverages be served in child care settings. The study also builds on existing evidence that non-CACFP sites and homes compared with CACFP sites and centers, respectively, are less likely to be fully adherent to nutrition policy. This suggests that additional resources to support full adherence, including monitoring and enforcement of existing policy, should emphasize providing milk and water at targeted sites. Implementation of a statewide child care beverage policy was feasible and has resulted in improved beverages being served to young children in child care centers and homes in California. Thus, adoption of child care beverage policy by other states is warranted.
